# Microbiota and Autism Spectrum Disorder

**Published:** 2019

**Authors:** Shahin Akhondzadeh

**Affiliations:** Psychiatric Research Center, Roozbeh Hospital, Tehran University of Medical Sciences, Tehran, Iran

Autism Spectrum Disorder (ASD) represents a neurodevelopmental condition characterized by two main deficits: impaired social communication and interaction; restricted and repetitive patterns of interests, behaviors or activities 1 with prevalence ranges from 2 to 20 per 1,000, worldwide 1,2. Presently, core symptoms of autism have no approved treatment. Autistic disorder management emphasis is on behavioral and educational modalities that target the core symptoms 3. Psychopharmacologic interventions are introduced to improve daily function and treat associated behavioral problems including hyperactivity, irritability, and aggression; leading to support the implementation of behavioral approaches through reducing the interfering symptoms 3.

In recent years, a growing number of studies have found evidence implicating dysregulation of immune responses and neuroinflammatory mechanisms in patients with ASD 3. Dysregulation of T helper cells 4, increased plasma levels of proinflammatory cytokines such as interleukin 1, 6 and 8 1–3, increased proliferation and activation of B cells and natural killer cells 4, decreased serum levels of immunoglobulin G and M 5 in presence of immunoglobulin G autoantibodies against neuron-axon filament and glial fibrillary acidic proteins 6, and increased microglial and astrocytic density and activation 5 have been documented.

Risperidone, as a serotonin 5-HT (2A) receptor antagonist that can attenuate dopamine release as well 3, is the only drug approved by the Food and Drug Administration (FDA) for the treatment of irritability in children with ASD. In recent years, research correlates risperidone use with significant weight gain 3 and with high rate of relapse after discontinuation of the medication in children with ASD 4. Despite this, due to lack of insight into the exact pathogenesis of ASD, no new medication could be approved as an adjuvant or standalone treatment for patients with ASD.

The fact that diet has a huge influence on our health should be common knowledge by now. But what research has been showing us in recent years is just how fundamental the influence of diet on our health can be. Surprising links between diet and a number of previously unsuspected diseases are being continuously established. But food does not affect us only after we are born, it actually starts to shape our health during pre-natal development. Many of these associations between diet and disease, including neurological diseases, are now known to be modulated by the gut microbiota. Research on the gut-brain axis is a blooming field where new and important findings keep streaming. Individuals with ASD often also have gastrointestinal problems and dysbiosis of the gut microbiota, being unclear whether an altered gut flora is a cause of a comorbidity of ASD. It has been suggested that changes in the gut microbiota may indeed play a role in the development of the behavioral symptoms associated with ASD, but the possible mechanisms of this link remain unknown 6.

As for autism, this link may come down to a particular molecule called interleukin-17a (or IL-17a), which is produced by the immune system. The molecule has already been associated with conditions like rheumatoid arthritis, multiple sclerosis, and psoriasis, and has been shown to serve an important role in preventing infections, notably those of the fungal kind. Importantly, it can also influence the way the brain develops in the womb.

The effects of the microbiome on the development of MIA-induced autism could be prevented either by modifying the pregnant mother’s microbiome, or by directly blocking IL-17a signaling.

Although ASD primarily impacts the brain, over recent years, links with other systems have become clear, in particular, gastrointestinal (issues seem to occur more often in individuals with ASD than in the rest of the population 6.
